# Warfarin anticoagulation control and risks of cerebral small vessel disease and cognitive impairment among elderly patients with ischemic stroke and atrial fibrillation: a retrospective cohort study

**DOI:** 10.3389/fnins.2026.2015432

**Published:** 2026-10-27

**Authors:** Dan Zhao, Wenping He, Zhifei Shen, Changyu Tan, Man Zhang

**Affiliations:** 1 Department of Pharmacy, Xuzhou First People's Hospital, Xuzhou, Jiangsu, China; 2 Department of Pharmacy, Xuzhou Maternity and Child Health Hospital, Xuzhou, Jiangsu, China

**Keywords:** atrial fibrillation, brain microbleeds, cerebral small vessel disease, ischemic stroke, population pharmacokinetics/pharmacodynamics, time in therapeutic range, vascular cognitive impairment, warfarin

## Abstract

**Objective:**

This study applied individual exposure metrics derived from population pharmacokinetic-pharmacodynamic (PopPK/PD) modelling to investigate the associations between warfarin anticoagulation quality, cerebral small vessel disease (CSVD) burden, and cognitive outcomes among older adults with ischemic stroke and non-valvular atrial fibrillation (NVAF).

**Methods:**

We included 300 patients aged ≥65 years with ischemic stroke and AF who had received warfarin for ≥3 months between January and December 2025. A nonlinear mixed-effects model was used to estimate individualized pharmacokinetic parameters and steady-state exposure metrics. Multivariable regression was performed to assess the associations of these metrics with CSVD burden, cognitive impairment, and the composite endpoint; the ability to identify high CSVD burden was compared between model-derived exposure metrics and conventional anticoagulation quality metrics, including time in therapeutic range (TTR) and international normalized ratio (INR) variability.

**Results:**

Steady-state average concentration (Cavg_ss_) was significantly positively associated with CSVD burden (per 100 ng/mL increment: OR = 1.32, *p* < 0.001), whereas TTR was not associated with burden (*p* = 0.444). High CSVD burden was significantly associated with cognitive impairment (Cox HR = 2.04, *p* = 0.007). The incidence of cognitive impairment increased stepwise across exposure tertiles (42.2%, 54.0%, and 75.4%, *p* < 0.001). The composite endpoint occurred significantly more often in patients with INR standard deviation (INR_SD) > 0.5 (*p* = 0.009). For identifying high CSVD burden, PopPK/PD-derived exposure parameters demonstrated differing within-cohort discrimination for identifying high CSVD burden: steady-state peak concentration (Cmaxss), Cavgss, and degree of fluctuation (DF) (AUC = 0.695, 0.679, and 0.651, respectively), with better discrimination compared with INR_SD and TTR (AUC = 0.557 and 0.493, respectively; *p* < 0.05).

**Conclusion:**

Within this cohort, PopPK/PD-derived individualized exposure parameters showed better discrimination for identifying patients with high CSVD burden compared with traditional TTR and INR variability, although discriminative ability remained modest for all markers, including those whose anticoagulation quality appeared adequate by conventional metrics. These findings provide a pharmacological foundation for refined anticoagulation quality assessment in this high-risk elderly population.

## 1 Introduction

Atrial fibrillation (AF) is highly prevalent in the elderly population and increases the risk of ischemic stroke by approximately five-fold, accounting for 15%–30% of all ischemic stroke cases (Scheitz et al., 2022). Patients with ischemic stroke complicated by AF exhibit a heavier overall disease burden, with significantly higher disability and recurrence rates than stroke patients without AF (Sposato et al., 2022). Anticoagulation therapy, as an independent protective factor against ischemic stroke recurrence and adverse prognosis, can substantially reduce the risks of thromboembolism and stroke recurrence (Jiang et al., 2025). However, less than half of such patients in China receive guideline-recommended anticoagulation treatment, resulting in severe insufficient anticoagulation. A major contributor to this clinical dilemma is the considerable difficulty in accurate anticoagulation quality assessment (Xu et al., 2021; Shen et al., 2018).

Warfarin is a classic and widely applied oral anticoagulant in clinical practice. Currently, the percentage of time in therapeutic range (TTR) is globally recommended to evaluate warfarin anticoagulation quality. Relevant guidelines have demonstrated that maintaining TTR above 65% is conducive to the prevention of ischemic stroke (Lip et al., 2018). Nevertheless, this assessment method requires repeated international normalized ratio (INR) monitoring and frequent dose adjustment, imposing a substantial burden on clinical management (Zhang et al., 2022). The population pharmacokinetic-pharmacodynamic (PopPK/PD) model is an emerging quantitative tool that integrates classical pharmacokinetic models with population statistical models. It enables quantitative analysis of population regularity, parameter distribution and influencing factors of *in vivo* drug processes. Accumulated studies have verified the favorable stability, goodness-of-fit and predictive performance of PopPK/PD models (Darwich et al., 2021; Marek et al., 2016)^.^

Existing PopPK/PD studies predominantly focus on model establishment and prediction accuracy verification. Limited studies have investigated the associations between PopPK/PD-derived quantitative anticoagulation indicators and long-term clinical outcomes in elderly patients with ischemic stroke complicated by non-valvular atrial fibrillation (NVAF) and cerebral small vessel disease (CSVD). Warfarin pharmacokinetics and pharmacodynamics are modified by CYP2C9 and VKORC1 genotypes, age, and body weight. Sole reliance on TTR cannot fully capture inter-individual variation in actual systemic anticoagulant exposure. It should be noted that anticoagulation-quality metrics (TTR, INR_SD), warfarin systemic-exposure parameters (Cavgss, Cmaxss, DF derived from PopPK/PD modelling), and CSVD-related risk represent distinct biological concepts. TTR quantifies the proportion of time INR falls within the therapeutic range, whereas PopPK/PD-derived parameters reflect in-vivo drug concentration magnitude and fluctuation. INR cannot fully capture inter-individual variation in actual systemic drug exposure, which provides a theoretical basis why pharmacokinetic exposure metrics may yield complementary information relevant to cerebral small-vessel injury. In this manuscript, we avoid directly labelling PopPK/PD-derived parameters as “anticoagulation quality indicators”. In this study, we built a warfarin PopPK/PD model incorporating genetic data for Chinese elderly patients to achieve precise individual-level quantification of anticoagulation exposure. We further explored associations between model-derived exposure parameters, CSVD burden, and cognitive outcomes. This work aims to supply pharmacological evidence supporting tailored anticoagulation management and cognitive protection for this vulnerable patient population.

## 2 Materials and methods

### 2.1 Baseline study population

This study was designed as a single-center retrospective cohort study. A total of 300 elderly patients diagnosed with ischemic stroke combined with NVAF who received inpatient treatment and outpatient follow-up at Xuzhou First People’s Hospital from January to December 2025 were consecutively enrolled, including 164 males and 136 females with a mean age of 76.00 ± 5.81 years. The inclusion criteria were as follows: (1) age ≥ 65 years; (2) the diagnosis of ischemic stroke was confirmed by cranial magnetic resonance imaging (MRI) or computed tomography (CT) in accordance with the Chinese Guidelines for the Diagnosis and Treatment of Acute Ischemic Stroke (Chinese Society of Neurology, Cerebrovascular Disease Group of Chinese Society of Neurology, 2024), accompanied by NVAF verified by electrocardiography or 24-h dynamic electrocardiography; (3) the duration of warfarin anticoagulation treatment was no less than 3 months with a relatively stable dosage; (4) complete follow-up data were available. The exclusion criteria were as follows: (1) patients with valvular atrial fibrillation or a history of mechanical or biological heart valve replacement; (2) patients who received other oral anticoagulants during warfarin treatment; (3) patients complicated with severe hepatic and renal insufficiency, uncontrolled hyperthyroidism or hypothyroidism, and other diseases that could significantly affect warfarin pharmacokinetics/pharmacodynamics or cognitive function; (4) patients with pre-diagnosed dementia or other neurodegenerative diseases, severe psychiatric disorders, and alcohol or drug dependence before enrollment; (5) patients with acute intracranial hemorrhage, brain tumors, craniocerebral trauma, central nervous system infection, or other lesions interfering with the imaging evaluation of CSVD. This study was approved by the hospital ethics committee (Approval No.: xyyll[2026]130).

### 2.2 Data collection

All clinical information was extracted systematically from the hospital electronic medical record system. Variables were grouped into eight categories:

- Demographic and anthropometric data: age, sex, years of formal education, body weight, body mass index (BMI).
- Comorbidities and stroke features: hypertension, diabetes mellitus, CHA₂DS₂-VASc score, HAS-BLED score, baseline National Institutes of Health Stroke Scale (NIHSS) score.
- Concomitant medications and genetic markers: amiodarone exposure, antiplatelet agents, CYP2C9 mutation carrier status, VKORC1-1639AA genotype.
- Laboratory indices: baseline INR, estimated glomerular filtration rate (eGFR), serum albumin.
- Baseline cognitive and emotional assessment: Montreal Cognitive Assessment (MoCA), 9-item Patient Health Questionnaire (PHQ-9), Trail Making Test Part A (TMT-A).
- Anticoagulation quality and PopPK/PD outputs: TTR, INR standard deviation (INR_SD), apparent clearance (CL), steady-state average plasma concentration (CavgssCavgss), steady-state peak plasma concentration (Cmaxss), degree of fluctuation (DF).
- CSVD imaging markers: Fazekas grading for periventricular and deep white-matter hyperintensities (WMH), total cerebral microbleed (CMB) count, lacunar infarct number.
- Follow-up endpoints: 6-month MoCA score, cognitive impairment (MoCA < 26), composite endpoints (intracranial haemorrhage, major bleeding, recurrent stroke/transient ischaemic attack [TIA], all-cause mortality).

### 2.3 Construction of PopPK/PD model and estimation of individual exposure parameters

A one-compartment model with first-order absorption was established for S-warfarin using the nonlinear mixed-effects model software (NONMEM 7.5, FOCE-I method). The time-concentration relationship was defined in Equation 1. Inter-individual variability was described using an exponential model (Pi = Ppop·e(ηᵢ)), while additive and proportional error models were applied to estimate residual errors. A stepwise covariate modeling approach (forward inclusion at *p* < 0.05 and backward elimination at *p* < 0.001) was adopted to evaluate the effects of age, body weight, eGFR, albumin, CYP2C9, and VKORC1 genotypes on CL and apparent volume of distribution (V). Continuous covariates were fitted with power functions, and categorical covariates were fitted with exponential functions. The covariate model for CL was presented in Equation 2. Model comparison was based on the objective function value (ΔOFV > 3.84, *p* < 0.05), with the Bayesian information criterion serving as an auxiliary judgment index.

$$C(t)=\frac{F\cdot \text{Dose}\cdot \mathrm{ka}}{\left(V\cdot (\mathrm{ka}-\mathrm{ke})\right)}\cdot (e^{-\mathrm{ke}\cdot t}-e^{-\mathrm{ka}\cdot t}),\mathrm{ke}=\frac{\mathrm{CL}}{V}$$ (1)

$$\begin{array}{l} CL_{i}=\theta_{\mathrm{CL}}\cdot {\frac{\mathrm{AG}E_{i}}{75}}^{\theta_{\mathrm{AGE}}}\cdot {\frac{\mathrm{eGF}R_{i}}{90}}^{\theta_{\text{eGFR}}}\cdot {\frac{\mathrm{AL}B_{i}}{40}}^{\theta_{\mathrm{ALB}}}\cdot {\frac{WT_{i}}{65}}^{\theta_{\mathrm{WT}}}\\ \cdot {\theta_{\mathrm{CYP}2C9}}^{\mathrm{MU}T_{i}}\cdot {\theta_{\text{VKORC}1}}^{AA_{i}}\cdot e^{\eta_{\mathrm{CL},i}} \end{array}$$ (2)

All covariates were centralized with typical values. MUTi and AAi were indicator variables for CYP2C9 loss-of-function allele carriage and VKORC1-1639AA genotype, respectively. *θ*_CYP2C9_ and *θ*_VKORC1_ represented the magnification coefficients of corresponding genotypes on CL, and *η_i_* obeyed the normal distribution of *N*(0, *ω*^2^) as inter-individual random effects. Based on the final model, individual CL and *V* values were estimated via Bayesian posterior estimation. Steady-state exposure indicators, including Cavgss (*τ* = 24 h), DF, and model-derived predictive fluctuation index (PredFluct), were calculated according to Equations 3–5. Model robustness was validated via 1,000 non-parametric bootstrap analyses (convergence rate > 90%, parameter bias < 15%) and visual predictive check (VPC).

$$\mathrm{Cav}g_{\mathrm{ss}}=\frac{\mathrm{AU}C_{\tau ,\mathrm{ss}}}{\tau },\mathrm{AU}C_{\tau ,\mathrm{ss}}=\frac{F\cdot \mathrm{Dos}e_{\mathrm{ss}}}{CL_{i}}$$ (3)

$$\mathrm{DF}=\frac{\left(\mathrm{Cma}x_{\mathrm{ss}}-\text{Ctroug}h_{\mathrm{ss}}\right)}{\mathrm{Cav}g_{\mathrm{ss}}}$$ (4)

$$\text{PredFluct}=\frac{\left(DF_{i}-\text{mean}(\mathrm{DF})\right)}{\left(\mathrm{SD}(\mathrm{DF})\right)}$$ (5)

PredFluct represents the standardized fluctuation index derived from individual DF values, used to quantify relative inter-patient variation in steady-state concentration fluctuation.

### 2.4 Anticoagulation quality evaluation and exposure stratification

The Rosendaal linear interpolation method was used to calculate TTR within the target INR range (Equation 6), assuming a linear change in INR values between two adjacent detections. INR_SD was calculated to reflect INR variability, and patients with INR_SD > 0.5 were defined as the high-variability group (Equation 7). All patients were stratified into low, medium, and high exposure groups according to the tertiles of Cavgss.

$$\mathrm{TTR}=\frac{\sum (\text{IN}R_{j}\in [\text{lower limit},\text{upper limit}]\times \Delta t_{j})}{\sum \Delta t_{j}}\times 100\%$$ (6)

$$\text{IN}R_{\mathrm{SD}}=\sqrt{\frac{\sum {\left(\text{IN}R_{j}-\text{IN}\bar{R}\right)}^{2}}{\left(n-1\right)}}$$ (7)

### 2.5 Outcome definition and evaluation

Three types of study outcomes were defined, including CSVD imaging burden, cognitive outcomes, and composite endpoints. Baseline cranial MRI/CT and baseline MoCA assessment were performed at admission before or at the initiation of warfarin therapy. All INR records used for PopPK/PD parameter estimation were collected during the initial ≥3-month warfarin treatment period. The 6-month MoCA cognitive assessment was performed approximately 6 months after warfarin commencement. (1) CSVD burden: The modified Fazekas scale was used to assess WMH. Susceptibility-weighted imaging (SWI) sequences were applied to count CMBs, lacunar infarcts, and enlarged perivascular spaces (EPVS). The total CSVD burden score ranged from 0 to 4 points, with scores < 3 defined as low burden and scores ≥ 3 defined as high burden. (2) Cognitive outcomes: Cognitive impairment was defined as a MoCA score < 26 at 6 months after warfarin initiation. The time origin for Cox survival analysis was set as the date of warfarin initiation, rather than hospital-enrolment date. Patients were retrospectively enrolled only when they had completed at least 3-month warfarin therapy after warfarin commencement. Baseline MoCA assessment was completed at admission close to warfarin initiation. In this study we applied a uniform MoCA < 26 cut-off without formal education-specific score correction; however, years of formal education was adjusted as a covariate in multivariate regression and Cox models. This threshold has been frequently adopted in previous studies focusing on Chinese elderly stroke patients. Lack of education-adjusted MoCA thresholds represents one limitation of our analysis. (3) Composite endpoints: The composite outcome included intracranial hemorrhage, major bleeding, recurrent stroke/TIA, and all-cause death during the follow-up period.

### 2.6 Statistical analysis

SPSS 26.0 software was used for all statistical analyses. Measurement data conforming to normal distribution were expressed as mean ± standard deviation, and independent samples t-test was used for inter-group comparison; data with non-normal distribution were presented as median and interquartile range, and the Mann–Whitney *U* test was adopted for group comparison. Count data were expressed as numbers and percentages (*n*, %), and the *χ*^2^ test was used for inter-group comparison. Multivariate logistic regression analysis was performed to analyze the associations between anticoagulation quality indicators and high CSVD burden, cognitive impairment, and composite endpoints, with confounding factors including age, sex, HAS-BLED score, CHA₂DS₂-VASc score, years of education, and baseline MoCA score adjusted. The Cox proportional hazards regression model was used to analyze cognitive outcomes. Receiver operating characteristic (ROC) curves were plotted to evaluate the predictive efficacy of each indicator for high CSVD burden, and the DeLong test was used to compare the area under the curve (AUC) values. A two-tailed *p* < 0.05 was considered statistically significant. The 100 ng/mL increment for Cavgss was pre-specified for regression analyses, chosen to represent a clinically meaningful magnitude of change in steady-state warfarin average concentration for ease of interpreting odds ratios.

## 3 Results

### 3.1 Baseline characteristics

A total of 300 enrolled patients were divided into the low CSVD burden group (*n* = 106) and the high CSVD burden group (*n* = 194) (Table 1). No statistically significant differences were observed between the two groups in terms of sex, years of education, body weight, hypertension, diabetes, HAS-BLED score, concomitant medications, CYP2C9/VKORC1 genotypes, and laboratory indicators (all *p* > 0.05). These variables were balanced between groups, whereas age, CHA₂DS₂-VASc scores and baseline cognitive function differed significantly. These imbalanced confounders were adjusted for in subsequent multivariable regression analyses.

**Table 1:** Baseline characteristics of enrolled patients.

Category	Variable	Low CSVD burden group (*n* = 106)	High CSVD burden group (*n* = 194)	*t*/*Z*/*χ*^2^	*p*
Demographic and anthropometric indicators	Age (years)	74.06 ± 5.53	77.06 ± 5.69	−4.452	<0.001
	Female, *n* (%)	45 (42.45)	91 (46.91)	0.384	0.536
	Years of education (years)	8.60 ± 4.94	8.71 ± 4.57	−0.221	0.826
	Body weight (kg)	63.78 ± 9.82	63.91 ± 9.88	−0.114	0.909
	BMI (kg/m^2^)	24.63 ± 3.88	25.02 ± 3.88	−0.83	0.407
Comorbidities and stroke characteristics	Hypertension, *n* (%)	62 (58.49)	133 (68.56)	2.627	0.105
	Diabetes, *n* (%)	40 (37.74)	92 (47.42)	2.232	0.135
	CHA₂DS₂-VASc score	4.08 ± 1.26	4.58 ± 1.31	−2.888	0.003
	HAS-BLED score	2.60 ± 0.90	2.64 ± 0.99	−0.363	0.705
	Baseline NIHSS score	4.89 ± 2.06	5.27 ± 2.15	−1.415	0.152
Concomitant medications and genotypes	Amiodarone use, *n* (%)	16 (15.09)	34 (17.53)	0.143	0.705
	Antiplatelet drug use, *n* (%)	27 (25.47)	63 (32.47)	1.284	0.257
	CYP2C9 mutation carriage, *n* (%)	12 (15.38)	16 (11.51)	0.367	0.545
	VKORC1-1639AA, *n* (%)	4 (5.13)	4 (2.88)	0.22	0.639
Laboratory indicators	Baseline INR	1.05 ± 0.10	1.05 ± 0.09	−0.524	0.601
	eGFR (mL/min)	78.40 ± 19.27	79.73 ± 17.84	−0.587	0.558
	Albumin (g/L)	37.58 ± 3.57	37.91 ± 3.80	−0.74	0.46
Baseline cognition and emotion	Baseline MoCA score	27.19 ± 2.56	25.52 ± 2.78	4.888	0
	PHQ-9	3.86 ± 3.33	6.05 ± 3.29	−3.782	0
	TMT-A (s)	52.57 ± 15.48	59.76 ± 16.00	−2.043	0.045
Anticoagulation quality and PopPK/PD parameters	TTR (%)	72.48 ± 15.42	71.22 ± 15.38	0.762	0.447
	INR_SD	0.56 ± 0.16	0.61 ± 0.19	−2.251	0.024
	CL (L/h)	0.24 ± 0.10	0.18 ± 0.09	5.158	0
	Cavg_ss_ (ng/mL)	318.96 ± 167.43	448.06 ± 249.13	−5.233	0
	Cmax_ss_ (ng/mL)	708.66 ± 322.76	997.64 ± 513.45	−5.71	0
	DF	0.62 ± 0.26	0.77 ± 0.26	−4.332	0
CSVD imaging characteristics	Fazekas periventricular score	0.50 ± 0.76	0.98 ± 0.98	−4.008	0
	Fazekas deep score	0.54 ± 0.69	1.06 ± 1.01	−4.066	0
	Positive WMH, *n* (%)	9 (8.49)	73 (37.63)	27.853	0
	Total CMBs number	0.38 ± 0.76	1.26 ± 0.98	−8.005	0
	Number of lacunar infarcts	0.51 ± 0.86	1.32 ± 1.04	−7.06	0
Follow-up outcomes	6-month MoCA	26.69 ± 2.94	23.19 ± 3.36	6.559	0
	Cognitive impairment (MoCA<26), *n* (%)	20 (18.87)	84 (43.30)	17.002	0
	Composite endpoints, *n* (%)	14 (13.21)	29 (14.95)	0.057	0.811

Compared with the low CSVD burden group, the high CSVD burden group presented significantly older age, higher CHA₂DS₂-VASc scores, and worse baseline cognitive function. Additionally, the high burden group exhibited significantly higher imaging burden indicators, including elevated positive rate of WMH, increased number of CMBs, and more lacunar infarcts (all *p* < 0.05). Notably, the conventional TTR indicator showed no significant difference between the two groups (*p* > 0.05), while all PopPK/PD model-derived exposure parameters differed significantly (*p* < 0.05), indicating that model-based exposure parameters were more sensitive to CSVD burden than traditional TTR. In terms of follow-up outcomes, the incidence of cognitive impairment was significantly higher in the high CSVD burden group (*p* < 0.05), whereas no significant inter-group difference was detected in the incidence of composite endpoints (*p* > 0.05).

### 3.2 PopPK/PD model parameters and performance

The final PopPK/PD model was determined as a one-compartment model with first-order absorption. The typical population clearance (*θ*_CL_) was 0.263 L/h, with an inter-individual variability (CV) of 26.3% and an ε-shrinkage value of 8.7%. The covariate model retained age, body weight, eGFR, albumin, CYP2C9 mutation, and VKORC1-1639AA genotype as significant influencing factors for CL, which was consistent with the Equation 2 established in the methodology. Advanced age, decreased eGFR, hypoalbuminemia, carriage of CYP2C9 loss-of-function alleles, and VKORC1-1639AA genotype significantly reduced CL and consequently elevated systemic warfarin exposure (Table 2). The coefficient of determination (*R*^2^) between model-predicted individual clearance values and observational values was 0.703. Among 1,000 bootstrap replicates, 998 runs achieved successful convergence, and the deviation between the median estimated parameters and original point estimates was less than 15%. These results demonstrated that the established warfarin PopPK/PD model was robust and reliable for individualized exposure parameter estimation.

**Table 2:** Parameters of the final PopPK/PD model.

Parameter	Estimate	95%CI	RSE%	*p*
*θ*_CL_ (L/h)	0.263	0.251–0.276	4.8	<0.001
*θ*_V_ (L)	10.2		6.5	<0.001
*θ*_ka_ (h^−1^)	1.28		12.3	<0.001
*ω^2^*_CL_	0.067		25.4	—
*ω^2^*_V_	0.089		31.2	—
*σ*_proportional_	12.40%		9.8	—
*σ*_additive_ (ng/mL)	22.6		14.5	—
ε-shrinkage	8.70%		—	—
*θ*_AGE_	−2.03	−2.535 to −1.565	12.3	<0.001
*θ*_WT_	0.69	0.499–0.901	16.4	<0.001
*θ*_eGFR_	1.15	0.970–1.325	7.8	<0.001
*θ*_ALB_	2.93	2.588–3.296	6.7	<0.001
*θ*_CYP2C9_	0.567	0.510–0.626	9.5	<0.001
*θ*_VKORC1-1639AA_	0.795	0.667–0.943	41.8	0.018

CL, clearance; *V*, apparent volume of distribution; ka, absorption rate constant; *θ*, typical structural model parameter; *ω*^2^, variance of inter-individual variability; *σ*, residual variability; ε-shrinkage, conditional shrinkage; *θ*_AGE_, *θ*_WT_, *θ*_eGFR_, and *θ*_ALB_ represent the effect exponents of continuous covariates; *θ*_CYP2C9_ and *θ*_VKORC1-1639AA_ represent the magnification coefficients of CL for patients carrying loss-of-function alleles and -1639AA genotype relative to wild-type genotypes, respectively.

The final established model was a one-compartment model with first-order absorption. The typical clearance value (θCL) was 0.263 L/h, the inter-individual variability of CL was 26.3%, and the conditional shrinkage was 8.7%. The covariate model retained age, body weight, eGFR, albumin, CYP2C9 mutation, and VKORC1-1639AA genotype as significant influencing factors for CL, which was consistent with Equation 2. Advanced age, decreased eGFR, hypoalbuminemia, carriage of CYP2C9 loss-of-function alleles, and VKORC1-1639AA genotype significantly reduced CL and increased warfarin exposure (Table 2). The coefficient of determination (*R*^2^) between individual predicted clearance values and observed values of the final model was 0.703. A total of 998 out of 1,000 bootstrap replicates converged successfully, and the deviation between the median parameter estimates and original point estimates was <15%. These findings demonstrated that the established model was robust and reliable for the estimation of individualized warfarin exposure parameters.

### 3.3 Associations of anticoagulation quality indicators with CSVD burden and cognitive outcomes

Multivariate regression analyses revealed that PopPK/PD-derived Cavgss exhibited the strongest correlation with clinical outcomes, whereas the conventional TTR indicator showed the weakest association (Table 3). In terms of CSVD burden, each 100 ng/mL increment in Cavgss increased the risk of high CSVD burden by approximately 30% (*p* < 0.001). INR_SD exhibited a weak but significant correlation with high CSVD burden (*p* = 0.021), while TTR showed no significant association with CSVD burden (*p* = 0.444). The proportion of high CSVD burden increased progressively across Cavgss tertiles (48%, 66%, 80%, *p* < 0.001, Figure 1a). For cognitive outcomes, high CSVD burden significantly elevated the risk of cognitive impairment (*p* < 0.05), and Cavgss was independently associated with cognitive impairment (*p* < 0.05). The incidence of cognitive impairment increased gradually across exposure tertiles (42.2%, 54.0%, 75.4%, *p* < 0.001, Figure 2). Regarding composite endpoints, patients with INR_SD > 0.5 had a significantly higher incidence of composite endpoints than those with low INR variability (*p* < 0.05). These findings indicated that the clinical value of INR variability was primarily reflected in composite endpoint prediction rather than CSVD burden identification.

**Table 3:** Associations of anticoagulation quality indicators with CSVD burden and cognitive outcomes.

Outcome	Indicator	Effect size	95%CI	*p*
High CSVD burden (Logistic regression)	Cavgss (per 100 ng/mL increase)	OR=1.32	1.13–1.55	<0.001
	TTR (per 10% increase)	OR = 1.11	0.85–1.46	0.444
	INR_SD (per 0.1 increase)	OR = 1.39	1.05–1.85	0.021
Cognitive impairment (Logistic regression)	High CSVD burden	OR = 3.44	1.46–8.14	0.005
	Cavgss (per 100 ng/mL increase)	OR = 1.55	1.18–2.02	0.001
	Cavgss tertile (trend test)	OR = 2.95	1.70–5.13	<0.001
	Cavgss tertile (high vs. low exposure)	OR = 8.75	2.87–26.67	<0.001
Cognitive impairment (Cox regression)	High CSVD burden	HR = 2.04	1.21–3.42	0.007
Composite endpoints (Logistic regression)	INR_SD>0.5	OR = 2.95	1.31–6.67	0.009

**Figure 1:**
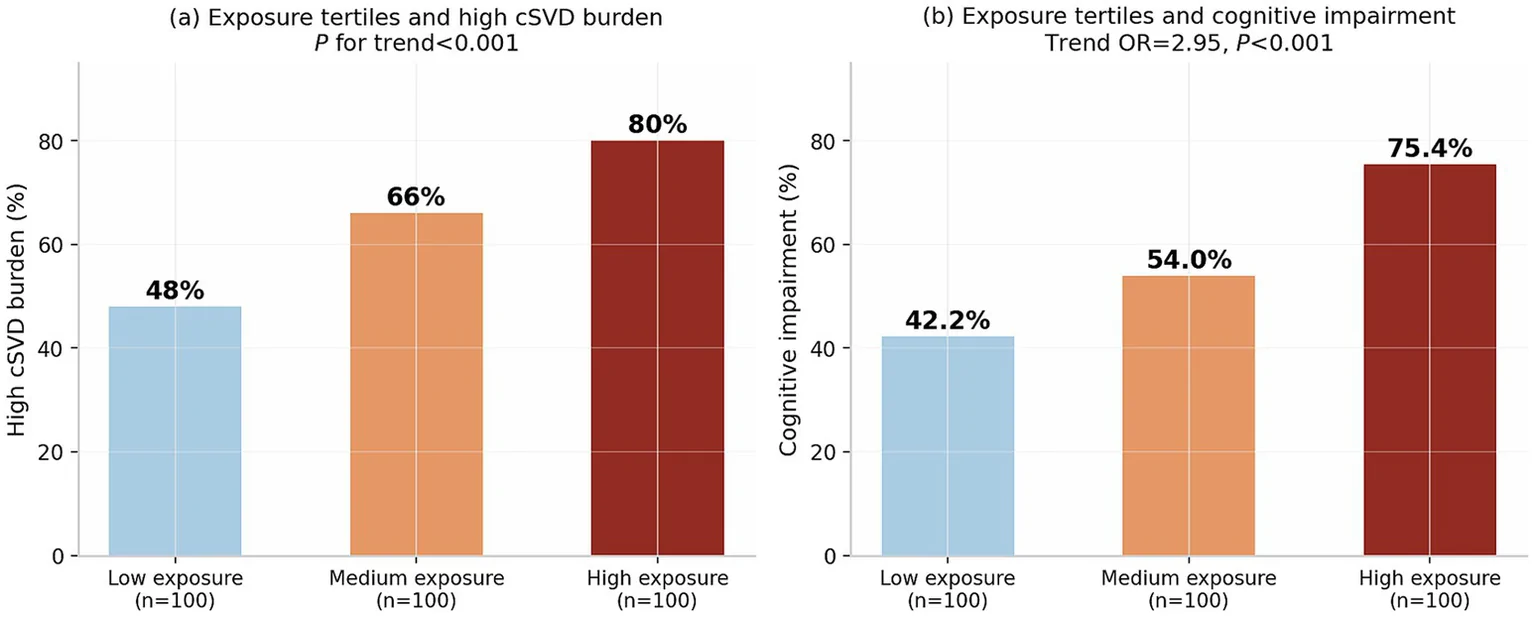
Dose–response gradient of clinical outcomes across steady-state Cavgss tertiles.

**Figure 2:**
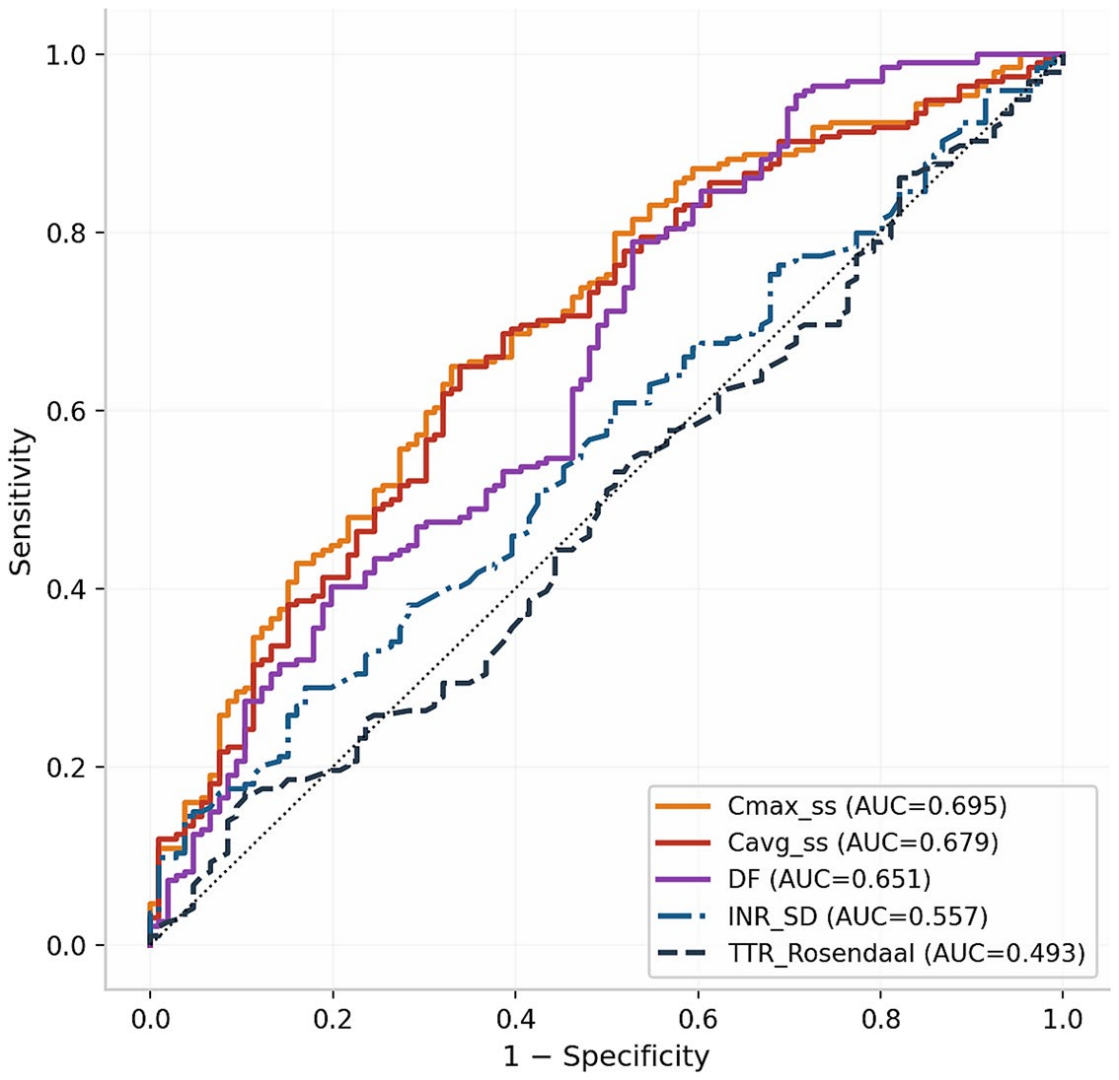
ROC curves of different indicators for identifying high CSVD burden.

### 3.4 Discrimination of PopPK/PD-derived exposure parameters for identifying high CSVD burden

ROC analyses demonstrated a distinct hierarchical gradient in the predictive performance of different anticoagulation indicators for high CSVD burden (Table 4; Figure 1). Conventional TTR exhibited nearly invalid predictive efficacy (AUC = 0.493), and INR variability showed moderate predictive ability (AUC = 0.557). In contrast, PopPK/PD-derived exposure parameters showed better discrimination within this cohortwith AUC values of 0.695 for Cmaxss, 0.679 for Cavgss, and 0.651 for DF. DeLong tests further confirmed that Cavgss presented significantly higher predictive accuracy than TTR (ΔAUC = 0.186, *p* < 0.001) and INR_SD (ΔAUC = 0.122, *p* = 0.011). These results indicated that the PopPK/PD model could effectively identify vulnerable patients with qualified TTR, acceptable INR variability, but excessive warfarin exposure and severe CSVD burden. Although INR_SD possessed certain predictive value, its clinical utility was mainly limited to composite endpoint prediction, with lower sensitivity for CSVD burden identification compared with model-derived individualized exposure parameters.

**Table 4:** ROC analysis of different indicators for predicting high CSVD burden.

Indicator	AUC	Lower bound of 95% CI	Upper bound of 95% CI
Cmax_ss_	0.695	0.631	0.756
Cavg_ss_	0.679	0.616	0.739
DF	0.651	0.584	0.716
INR_SD	0.557	0.494	0.623
TTR_Rosendaal	0.493	0.422	0.561

## 4 Discussion

Cerebral small vessel disease (CSVD) is a crucial contributor to vascular cognitive impairment and even dementia, and its characteristic imaging and pathological alterations mainly include cerebral white matter lesions, lacunar infarcts, and cerebral microbleeds (Chojdak-Łukasiewicz et al., 2021). Previous clinical studies have indicated that approximately 45% of all dementia cases are attributable to or closely associated with CSVD, and this disease burden increases progressively with aging in the elderly population (Elahi et al., 2023). From a neuroscientific mechanistic perspective, the pathophysiological basis of CSVD-related cognitive impairment is not limited to the simple accumulation of imaging biomarkers. Instead, it involves a cascade of pathological processes, including blood–brain barrier disruption, endothelial dysfunction, chronic cerebral hypoperfusion, and neuroinflammation (Gao et al., 2022; Zhang et al., 2025). Notably, this evidence suggests that the protective effects of anticoagulation therapy on cerebral outcomes are far beyond the reduction of thromboembolic events. We emphasize the conceptual distinction between exposure magnitude and exposure stability. Stable, appropriately-dosed anticoagulant exposure within the therapeutic range may be correlated with alleviated cerebral microvascular injury and blood–brain barrier leakage, which could be linked to slower CSVD progression; by contrast, excessively high systemic warfarin exposure (reflected by elevated Cavgss in our data) was associated with greater CSVD burden and higher rates of subsequent cognitive impairment (Gao et al., 2022).

The present study performed individualized warfarin exposure evaluation based on a PopPK/PD model in 300 elderly patients with ischemic stroke complicated with non-valvular atrial fibrillation, and further explored the correlations between model-derived exposure parameters, CSVD burden, and cognitive outcomes. The results demonstrated that PopPK/PD-derived exposure parameters exhibited a distinct hierarchical advantage in identifying high CSVD burden, with AUC values ranging from 0.65 to 0.70 for Cmaxss, Cavgss, and DF, which were significantly superior to the predictive efficacy of INR variability (AUC = 0.557) and conventional TTR (AUC = 0.493). These findings indicate that traditional anticoagulation quality assessment systems dominated by TTR and INR variability have inherent limitations in identifying subclinical cerebral organ damage represented by CSVD (Zhou et al., 2023). The core advantage of the PopPK/PD model lies in its ability to integrate multiple individual factors, including CYP2C9/VKORC1 genotypes, age, and body weight, to restore the real steady-state warfarin exposure and fluctuation characteristics of individual patients, thus achieving more accurate quantification of actual anticoagulation intensity (Zhou et al., 2020; Guan et al., 2016). In contrast, TTR and INR variability only reflect anticoagulation quality from two single dimensions, namely the time within the therapeutic range and the discrete degree of INR values, and fail to capture essential inter-individual differences in steady-state drug exposure levels (Ciurus et al., 2015; Ebrahim et al., 2018), which explains their poor sensitivity for identifying elevated CSVD burden.

Further stratified analysis revealed that high CSVD burden increased the risk of cognitive impairment by more than two-fold (HR = 2.04), and this adverse outcome was independently correlated with warfarin steady-state exposure levels. Pathophysiologically, CSVD acts as a critical pathological foundation for vascular cognitive impairment and dementia, and the gradual accumulation of white matter lesions, lacunar infarcts, and cerebral microbleeds is the key pathological process driving cognitive decline. The findings of the present study are highly consistent with this established pathological mechanism (Mirzai et al., 2026; Luo et al., 2026). Furthermore, this study suggests that adequate and stable warfarin exposure may help delay CSVD progression and protect cognitive function in elderly patients with atrial fibrillation (Ko et al., 2017). In addition, although INR variability (INR_SD > 0.5) showed moderate efficacy in identifying CSVD burden, it was significantly correlated with the occurrence of composite endpoint events (OR = 2.95). This finding confirms that the clinical value of INR variability is primarily reflected in the prediction of long-term prognostic endpoints rather than the identification of subclinical cerebral microvascular damage (Casais et al., 1999; Mansour et al., 2019). Collectively, different anticoagulation quality indicators possess distinct clinical evaluation dimensions. Model-derived steady-state exposure levels better reflect the cumulative effect of chronic cerebral organ damage, whereas excessive INR fluctuation predominantly indicates unstable anticoagulation status, which is closely associated with increased risks of thromboembolism and bleeding events (Mansour et al., 2019).

In summary, this study verifies that PopPK/PD model-based quantitative evaluation of warfarin anticoagulation quality is superior to traditional TTR and INR variability in identifying high-risk CSVD burden and subsequent cognitive impairment in elderly patients with ischemic stroke and atrial fibrillation. The present findings provide novel evidence-based support for refined individualized anticoagulation management and cognitive protection in this specific patient population. Beyond guiding individualized warfarin dosage adjustment, PopPK/PD-derived exposure parameters provide quantifiable biomarkers for identifying cerebral microvascular damage and long-term cognitive outcomes in this cohort. Nevertheless, this study is a single-center retrospective cohort study with inherent limitations. Large-sample, prospective multicenter studies are required in the future to further validate the clinical practicality and generalizability of the PopPK/PD model in anticoagulation management and cerebral prognosis prediction.

## Data Availability

The raw data supporting the conclusions of this article will be made available by the authors, without undue reservation.
